# Supplementary material for: Causal Relationship between Aging and Anorexia Nervosa: A White-Matter-Microstructure-Mediated Mendelian Randomization Analysis
Source: Biomedicines. 2024 Aug 16;12(8):1874. doi: 10.3390/biomedicines12081874 (PMC11351342; doi:10.3390/biomedicines12081874)
Supplement: Supplementary file 1 [file biomedicines-12-01874-s001.zip › Supplementary Figure/Figure S8/Leaveoneout plot of MR analysis for TL to ubm-b-2067.pdf]

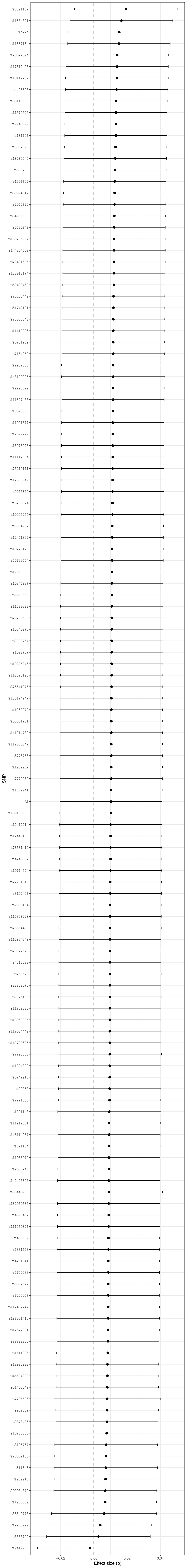
rs3891167
rs11584821
rs4724
rs11557154
rs28577594
rs117512405
rs10112752
rs4498805
rs80116508
rs11579626
rs9940099
rs131797
rs6007020
rs13230646
rs869785
rs1907702
rs80324517
rs2056726
rs34550383
rs6590343
rs139795227
rs144204502
rs78491606
rs188918174
rs59409453
rs76666449
rs61748181
rs76065543
rs11412296
rs6751209
rs7164950
rs2967355
rs143190905
rs2293579
rs111527438
rs3093888
rs11991877
rs7099229
rs16978028
rs11117354
rs76219171
rs17803849
rs9955360
rs3785074
rs10905255
rs6054257
rs12451892
rs10773176
rs56799554
rs12369950
rs10845387
rs6669563
rs11699829
rs73730598
rs10840270
rs2282764
rs1023767
rs10805346
rs113525195
rs376641875
rs185174247
rs41269079
rs56061761
rs141214782
rs117630647
rs6776756
rs1957937
rs7772289
rs1332941
All
rs150150565
rs12412214
rs17445108
rs73581419
rs4743037
rs10774624
rs77231040
rs8102497
rs2555104
rs116863223
rs75664430
rs112394943
rs79977579
rs4616688
rs762679
rs28363070
rs2276182
rs11769630
rs13062095
rs117034449
rs142730696
rs7790856
rs41304832
rs5742915
rs429358
rs7221585
rs1291143
rs11212631
rs145114957
rs871134
rs11085072
rs2538745
rs142426306
rs35446936
rs182059586
rs4695407
rs111950327
rs450962
rs6881568
rs4731541
rs6790988
rs6587577
rs7209057
rs117407747
rs137901416
rs17677991
rs77732866
rs1611236
rs12925933
rs45604339
rs61405042
rs7705526
rs932002
rs9878436
rs10768683
rs8105767
rs28502153
rs611646
rs939916
rs202034370
rs1985369
rs35640778
rs2763979
rs6536702
rs9419958
SNP
−0.02
0.00
0.02
0.04
Effect size (b)
